# Stemness of Cancer: A Study of Triple-negative Breast Cancer From a Neuroscience Perspective

**DOI:** 10.1007/s12015-024-10809-0

**Published:** 2024-11-12

**Authors:** Mustafa B. A. Djamgoz

**Affiliations:** Department of Life Sciences, Imperial College London, South Kensington Campus, London, SW7 2AZ UK

**Keywords:** Oncofoetal, Hypoxia, Drug resistance, Ion channel, Splicing, Autoantibody, Neuroscience, Antibody, Tumour microenvironment, Epigenetics, Bioelectricity, Diagnostics, Electrotherapy

## Abstract

Stemness, giving cancer cells massive plasticity enabling them to survive in dynamic (e.g. hypoxic) environments and become resistant to treatment, especially chemotherapy, is an important property of aggressive tumours. Here, we review some essentials of cancer stemness focusing on triple-negative breast cancer (TNBC), the most aggressive form of all breast cancers. TNBC cells express a range of genes and mechanisms associated with stemness, including the fundamental four “Yamanaka factors”. Most of the evidence concerns the transcription factor / oncogene *c-Myc* and an interesting case is the expression of the neonatal splice variant of voltage-gated sodium channel subtype Nav1.5. On the whole, measures that reduce the stemness make cancer cells less aggressive, reducing their invasive/metastatic potential and increasing/restoring their chemosensitivity. Such measures include gene silencing techniques, epigenetic therapies as well as novel approaches like optogenetics aiming to modulate the plasma membrane voltage. Indeed, simply hyperpolarizing their membrane potential can make stem cells differentiate. Finally, we give an overview of the clinical aspects and exploitation of cancer/TNBC stemness, including diagnostics and therapeutics. In particular, personalised mRNA-based therapies and mechanistically meaningful combinations are promising and the emerging discipline of ‘cancer neuroscience’ is providing novel insights to both fundamental issues and clinical applications.

**Graphical Abstract:**

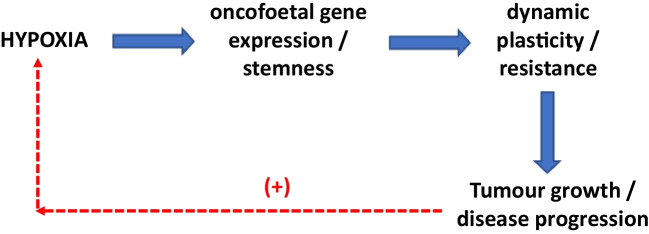

## General Introduction

Cancer now is one of the most common causes of death with 1 in 2–3 people diagnosed with the disease in some form during their lifetime. The annual number of cancer diagnoses worldwide is expected to rise to some 28 million, while the number of cancer-related deaths to 16 million by 2040 [1]. In all solid tumours, except gliomas, a prominent cause of death is metastasis, the complex process in which cancer cells escaping from primary tumours ultimately invade secondary sites giving rise to organ failure. Many problems remain in clinical management of cancer, especially difficulty of early, functional diagnosis and long-term effective therapy without undesirable side effects. Regarding the latter, the situation is worse for metastatic disease since it often necessitates systemic treatment with cytotoxic drugs to which resistance then develops over time.

Cancer cells/tissues demonstrate varying degrees of dedifferentiation, and many genes therein are (re)expressed in their embryonic (“oncofoetal”) forms [e.g. 2]. Thus, cancer cells have inherent stemness and, consequently, tumours are heterogenous in their cellular make-up. An obvious case of dedifferentiation / stemness can be seen in a type of germ-cell cancer called “teratocarcinoma”. These are rare tumours the embryonic nature of which can result in differentiation into different types of adult tissue such as bone, teeth, muscle and hair [3]. On the whole, the degree of dedifferentiation is positively correlated with the aggressiveness of the tumour, but this may vary from cancer to cancer [e.g. 4].

It is not clear why de-differentiation occurs in cancer. One possibility is that it is a response to the pathological change and the body’s attempt to reverse the ‘damage’ by reverting the cancer tissue back its embryonic form, so it can rebuild itself normally. A similar phenomenon of embryonic gene (re)expression occurs following myocardial infarction [e.g. 5] and wounding [6]. In fact, oncofoetal gene expression could be a general response to pathological insult. Interestingly, this phenomenon is also associated with hypoxia which occurs widely in 90% of growing solid tumours because of blood supply and oxygenation being impaired. Overall, therefore, the following chain of events involving stemness can occur in cancer: *Hypoxia* > *oncofoetal gene expression / stemness* > *dynamic plasticity / resistance* > *tumour growth / disease progression* (with possible positive feedback linking growth back to hypoxia). Thus, exposure to hypoxic conditions generally make tumours more aggressive by clonal selection [e.g. 7]. Overall adaptation to hypoxic environments involve induced tolerance to immune surveillance, chemotherapy and enhanced radiation tolerance [8].

Stem cells, including cancer stem cells (CSCs) are capable of self-renewal and plasticity. The latter is a broad term highlighting the fact that such cells can change their molecular make-up and functional characteristics in space and time. This happens transcriptionally and post-transcriptionally in response to the biochemical and biophysical conditions encountered dynamically at various stages of the cancer process. Thus, plasticity manifests itself during cancer progression, as cells become exposed to different micro-environments, as well as in response to treatment. Whilst CSCs have intrinsic plasticity, however, plasticity does not ensure stemness. Plasticity gives CSCs multi-lineage potential and can enable them to become drug resistant following treatment [9]. In addition, CSCs can possess intrinsic resistance mechanisms including expression of *MDR1*, a multi-drug transporter / resistance gene [10]. Consequently, during treatment (e.g. chemotherapy) tumours enhance their stemness, qualitatively and quantitatively and, in the process, become increasingly drug resistant. This makes effective treatment more and more difficult ultimately jeopardising patient output. Importantly, however, the process appears reversible, at least partially, and once treatment is paused, CSCs may re-differentiate [11].

In conclusion, stemness or more broadly plasticity is a major hurdle to successful management of cancer. The cell-of-origin (the cell type from which a given tumour originates) plays a crucial role in determining the properties of CSCs and their contribution to tumour heterogeneity. Here, we investigate the problem of CSCs in the context of ‘triple-negative breast cancer’ (TNBC), adopted as a study model with supporting novel insight derived from neuroscience. Data from other solid tumours are used for completeness.

## Stemness of TNBC

### An Overview

TNBC is the most aggressive form of breast cancer [12]. It is characterised basically by the lack of expression of oestrogen and progesterone receptors as well as the human epidermal growth factor receptor 2 (HER2), altogether accounting for 15–20% of all breast cancer cases. As a result, it is the hardest of the all breast cancers to treat, responsible for ca. 25% of deaths. The standard procedure is combination of surgery with chemotherapy. TNBC tumours are some 3-fold more likely to metastasize within 5 years of diagnosis [12]. Consequently, median time to death, presumably from metastatic disease, is significantly shorter (ca. 4.2 vs. 6 years) and survival is poorer for TNBC compared to other breast cancers [13].

**Fig. 1:**
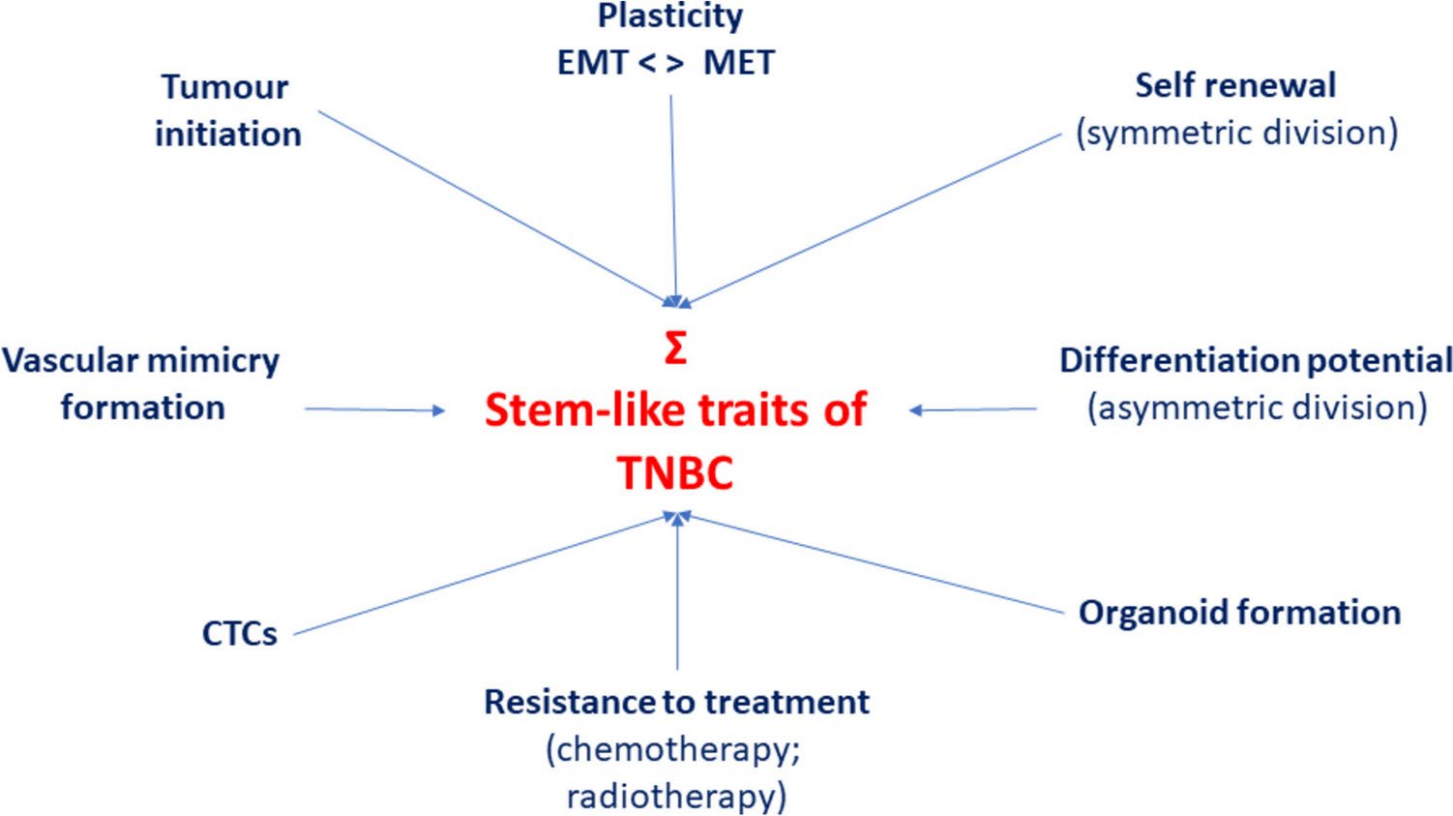
Traits of triple-negative breast cancer (TNBC) that give it stemness. Several characteristics that contribute to stemness or follow from it are highlighted. “Σ” indicates their summation. EMT, epithelial-mesenchymal transition; MET, mesenchymal-epithelial transition (reverse of EMT); CTC, circulating tumour cell. Contents adopted from Huang et al. [16]

Overall, TNBC tumours are less differentiated and, correspondingly, more enriched with CSCs compared to non-TNBC breast cancers [14]. The stemness of TNBC contributes to its poor clinical outcomes so elimination of CSCs promises to suppress the progression and recurrence of the disease [15]. The various attributes of TNBC normally associated with stem cells are summarised in Fig. 1. These characteristics are driven by a wide range of molecular and signalling mechanisms that are spatially and temporally highly dynamic and organized hierarchically in nucleus, cytoplasm and plasma membrane. Of the subtypes of TNBC, those with heightened stemness-related pathways, e.g. TGF-β signalling, Wnt pathway and epithelial-mesenchymal transition (EMT), have relatively higher proliferation-related gene expression levels and poorer prognosis [16].

In this review, we highlight some of the progress made in this field of TNBC stemness, focusing mainly on evidence presented over the last 5 years.

### Biomarkers and Mechanisms of Stemness

Four primary transcription factors (Oct3/4, Sox2, Klf4 and c-Myc), known as the “Yamanaka factors”, are highly expressed in embryonic stem cells (ESCs), and their over-expression, independently or in synergic combination, can induce pluripotency in human somatic cells [17]. Several studies have characterised in considerable detail the Yamanaka factors as well as several other genes and their associated signalling mechanisms in relation to the stemness of TNBC in vitro and in vivo [14].

Inhibiting MCL1, an antiapoptotic protein, in TNBC cells downregulated stemness makers including *Oct3/4* and *Sox2*, and suppressed invasiveness [18]. Conversely, promoting *Sox2*/*Oct4* response elements by activating oestrogen-β4 receptors enhanced TNBC stemness and this led to reduced sensitivity to chemotherapy [19]. The long noncoding RNA (lncRNA) TGFB2-AS1 was identified as a reversible inhibitory regulator of TNBC stemness, significantly decreasing tumorigenicity and lung metastasis in vivo [20]. Correspondingly, combined prognostic analysis of TGFB2-AS1 and TGFβ2 in TNBC patients showed that high TGFB2-AS1 and low TGFβ2 levels correlated with better outcome. Another lncRNA (LUCAT1) formed a positive feedback loop involving *Sox2* that, also, promoted stemness in TNBC [21]. Upregulation of yet another lncRNA, DANCR, induced by the acidic protein TUFT1, promoted metastasis via the miR-874-3p-Sox2 axis [22]. *Sox2* expression in TNBC was further controlled by miR-574-5p [23] and miR-708 [24].

Thus, a given transcription factor, such as *Sox2*, could be involved in multiple pathways in TNBC emphasising the multiplicity of the control mechanisms regulating the stemness, consistent with its powerful role in the cancer process.

Roberts et al. showed that TNBC cells express Klf4, albeit at low levels, and this functions as a major inhibitory controller of EGFR expression and activity [25]. Thus, Klf4 repressed transcription of *EGFR*, leading to reduced levels of total and activated/phosphorylated form of EGFR, and downstream signalling. Indeed, EGFR suppression proved necessary for Klf4 to inhibit TNBC aggressiveness. Importantly, Klf4 also controlled the sensitivity of TNBC cells to erlotinib, an FDA-approved inhibitor of EGFR. Nakajima et al. confirmed that Klf4 is expressed TNBC cells and showed that it would induce p53-independent apoptosis [26]. The lncRNA CCAT2 was highly expressed specially in TNBC in vitro and in vivo, promoted the expression of stemness markers including Oct4, Nanog and Klf4, and increased mammosphere formation [27].

From this limited account, it would appear that although Klf4 is an essential stemness (“Yamanaka”) factor, its contribution to TNBC aggressiveness can be bidirectional, i.e. it could function, at least in part, as a compensatory mechanism in control of stemness. This would also emphasise the diversity of mechanisms controlling TNBC stemness, but further work is required to test this hypothesis.

**Fig. 2:**
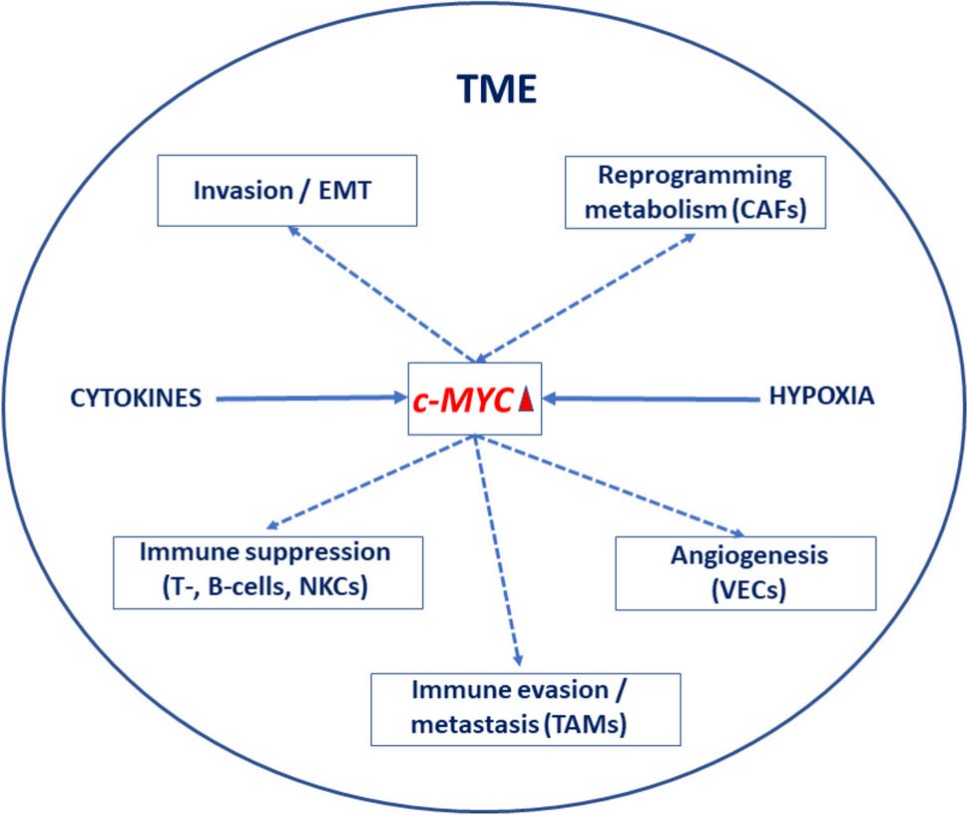
c-Myc-mediated cellular interactions within the tumour microenvironment (TME). Involvement of c-Myc in breast cancer stem cell behaviour and in the crosstalk with cells of the TME. c-Myc promotes epithelial-mesenchymal transition (EMT) leading to invasiveness, regulates angiogenic activity of vascular endothelial cells (VECs), and functioning of tumour-associated fibroblasts (CAFs) in two-way interactions. Furthermore, c-Myc is able to modulate cells of the immune system, including tumour-associated macrophages (TAMs), natural killer (NK) cells, and T- and B-lymphocytes, as well as expression of PD-L1 and CD47. Thus, it promotes immune evasion and immunosuppression. In turn, TME can impact c-Myc expression via hypoxia (HIF-2α), and several agents including growth factors and various cytokines released from CAFs. Based on information from Gao et al. [28] where further details can be found

**Fig. 3:**
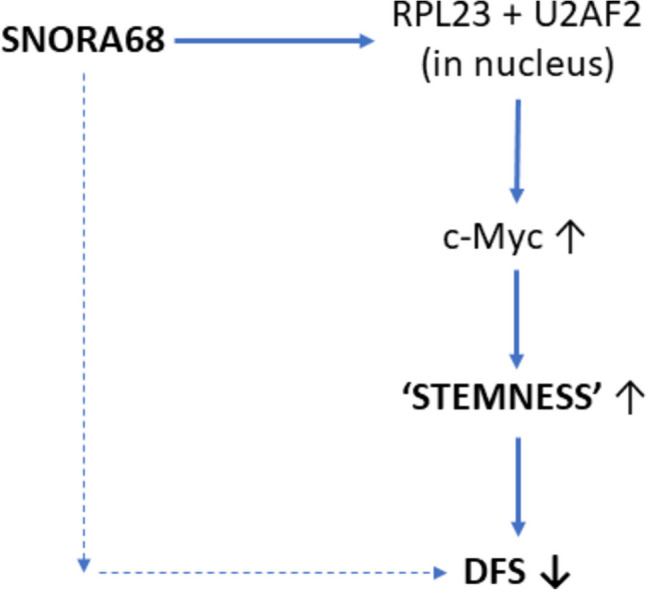
Control of *c-Myc* expression in TNBC. The mechanism by which SNORA68 promotes stemness of TNBC and leads to reduced disease-free survival (DFS). SNORA68 with U2AF2 retains RPL23 in the nucleolus and reduces the RPL23-mediated decrease in *c-Myc* expression (in the nucleoplasm). Thus, *c-Myc* expression is upregulated, leading to enhanced stemness. Upward and downward pointing arrows on the side denote increasing and decreasing effects, respectively. The dotted lines indicate the correlation between the plasma SNORA68 level and DFS. Derived from data in Zhang W et al. [29]

Most work has been done on *c-Myc* in TNBC where it is highly expressed and plays a central role in a range of cellular behaviours and interactions within the tumour microenvironment (TME) (Fig. 2) [28]. In fact, this is the most aggressive of the oncogenes. A recent study probed the possible involvement of *c-Myc* in the role of small nucleolar RNAs (snoRNAs) in control of TNBC stemness [29]. TNBC expressed high levels of SNORA68 which correlated with tumour size, Ki-67 level and TNM stage. Functionally, SNORA68 promoted the cellular stemness and carcinogenesis of TNBC in vitro and in vivo (xenograft model). Mechanistically, raised SNORA68 expression led to increased nucleolar RPL23 expression retained by binding U2AF2 (Fig. 3). This led to upregulation of *c-Myc* expression as the basis of the stemness. At patient level, correspondingly, plasma SNORA68 concentration was inversely correlated significantly with disease-free survival. Elsewhere, the secretory-carrier-membrane-protein 3 (SCAMP3) was shown to promote TNBC stemness and progression (cell proliferation, clonogenicity, tumour spheroid formation and migration in vitro and tumour growth in vivo) through *c-Myc* [30]. Ubiquitin-specific protease 22 (USP22) enhanced the rate of extracellular acidification, proliferation, spheroid number, CD44^+^/CD24^−^ cell number, and the expression of stemness genes and EMT-related markers. These effects involved de-ubiquitination and stabilization of *c-Myc* [31]. The atypical cyclin-like protein Spy1 was found to be highly expressed in human TNBC cell and patient samples and correlated with c-Myc protein levels [32]. Furthermore, silencing Spy1 greatly increased response to chemotherapy (cyclophosphamide—taxol combination). c-Myc expression in TNBC has also been shown to be controlled by several miRNAs [33, 34]. This interaction is complex and two-way, however, since silencing *c-Myc* affected the expression of 126 different miRNAs (84 upregulated, 42 downregulated) [33]. Particularly interesting was miR-4723-5p the target gene of which (*TRAF4*) was involved in metastasis. Overall, this is yet another example of the mechanistic multiplicity controlling TNBC stemness.

As regards non-Yamanaka factors, the messenger RNA (mRNA) expression-based stemness index (mRNAsi) was used to quantify the unique characteristics of CSCs in 127 patients of TNBC [35]. The role of the stemness was analysed in relation to clinical characteristics using several different bioinformatics algorithms. Association analysis showed that mRNAsi values were significantly higher in stage I/II versus stage III/IV disease. Based on the median mRNAsi value, the patients were divided into high and low mRNAsi groups, which revealed some interesting insights. First, the high-mRNAsi group had poor survival compared with the low-mRNAsi cases. Second, there was a notable negative correlation between mRNAsi and the immune score, i.e. the low-mRNAsi group had significantly higher immune activity, indicating that CSCs may promote TNBC development by weakened immune function. Third, the differentially expressed genes (DEGs) were related to mitotic nuclear division, regulation of mitotic nuclear division, and enriched in cancer-promoting pathways, including PI3K-Akt, TGF-β and MAPK, consistent with CSCs accelerating cancer progression. The patients were reclassified into two stemness subgroups: stemness subtypes I and II with the latter having higher enrichment of immune cells and immune infiltration. The expression levels of CD80, CD86 and the value of ‘tumour mutation burden’ (TMB) were higher in subtype I vs. II. A prognostic risk model was constructed based on the stemness of tumours from TNBC patients [35]. Analyses of Kaplan-Meir and receiver-operator characteristics confirmed that the patients in the ‘low-risk’ group had significantly longer overall survival times compared to the ‘high-risk’ group (hazard ratio > 1). The following genes were highly expressed in the ‘high-risk’ group: *STMN2, SCGB2A2, RUNDC3B, PCDHGA3, IL1RL1, BMP4, CCBE1, CELSR3* and *GPRC5C*. This work suggested that clinical targets / treatments can be personalised for TNBC patients based on the stemness characteristics of the tumours (see the “Clinical Aspects” section).

Ring et al. focused on the E1A-associated protein p300 (EP300) which functions as histone acetyltransferase that regulates transcription via chromatin remodelling [36]

## A Neuroscience Approach

An intriguing recent development in oncology is what has come to be called “cancer neuroscience”, i.e. understanding and treating cancer using neuroscience concepts and techniques, exploiting the vast knowledge that has been accumulating since the 1990s [37]. There are two major facets of cancer neuroscience– 1) innervation of tumours and (2) expression of voltage-gated ion channels and ionotropic neurotransmitter receptors normally associated with neurones and other ‘excitable’ cells [37–39]. First, the nerve input can associate with several of the hallmarks of cancer [40]. A recent study has shown that breast cancer indeed exploits novel neural signalling pathways for bone-to-meninges metastasis [41]. It is also possible that the neuronal impact on the TME can influence stemness and, ultimately, disease progression and survival [42]. Thus, serotonin produced by enteric neurons was shown to initiate colorectal cancer stem cell self-renewal and tumorigenesis [43]. Second, of the neuronal proteins expressed de novo in carcinomas, some of the ion channels arise as neonatal splice variants, consistent with the common dedifferentiation process. In fact, many cancer drivers end up in causing splice defects [44]. This is seen most clearly for the voltage-gated sodium channel (VGSC) subtype Nav1.5 which is dominant in the TNBC model cell line MDA-MB-231 (as well as in colon cancer) as an embryonic splice variant [39, 45–48]. nNav1.5 mRNA and protein expression also occur in vivo and correlate with metastatic status [45]. Neonatal Nav1.5 (nNav1.5) is pharmacologically distinct from the adult form of the channel (in part due to the aspartate-lysine double-charge switch) and can also readily be targeted using an antibody [45, 49–51]. Further clinical significance and potential of nNav1.5 in TNBC are discussed in the “Immune Aspects and Biologics” section. More broadly, bioelectric signalling, driven by ion channels and neurotransmitters, inherent to neurones, also occurs in stem cells and can exert significant control on the differentiation process [52]. These include mechanosensitive ion channels which probably manifest themselves during location-specific differentiation within the scope of cancer cell plasticity [53, 54]. The therapeutic potential of stem cell / TNBC bioelectricity are discussed in the “Electroactive Agents and Electrotherapy” section.

## Clinical Aspects

Since stemness plays a major role in progression of cancer, including TNBC, its underlying mechanisms could serve as targets for the clinical management of the disease.

### Diagnostics

The degree of tissue differentiation is already used as a light microscopic diagnostic tool in histopathological assessment of tumours [55]. As acquisition of stemness accelerates cancer progression, such diagnosis can serve as an early indicator of disease, a primary requisite for an expedient diagnostic biomarker. Several established and emerging CSC markers are shown in Table 1. Whilst the evidence for some of these markers (e.g. CD44) seem solid and consistent, others are still short of full validation. A commonly used albeit rather non-specific indicator is “carcinoembryonic antigen” (CEA) protein readily detectable in blood.

**Table 1:** Representative biomarkers of cancer stem cells (CSCs), except Yamanaka factors, in common cancer types. Modified and extended from Saito et al. [56]

Cancer Type	Marker(s) of CSCs	Additional reference(s)
Breast (TNBC)	CD44/CD24^−^, CD133, ALDH1, integrin alpha 6, EpCAM, Cadherin 3, HER2, prominin-1, CXCR1	Ali et al. [57]
Prostate	CD44, CD133, integrin alpha 6	Harris & Kerr [58]
Lung	CD133, ALDH1, CD44	Zheng Y et al. [59]
Pancreatic	CD133, CXCR4, SSEA-1, CD44, ALDH1	Bubin et al. [60]
Colorectal	EpCAM, CD44, CD24 CEA-CAM, CDX1	Omran et al. [61]
Melanoma	CD271, CD20, CD133, ABCB5	Parmiani [62]
Ovarian	CD133, CD44, CD24, CD117, ALDH1	Królewska-Daszczyńska et al. [63]
Cervical	CD44, CD133	Mendoza‑Almanza et al. [64]
Gastric	CD44, HER2, APC, p53, kRAS, PTEN, LGR5, CCKBR, RHOA, CDH-1, SMAD5, ATP4B, PGA3	Wang J et al. [65]

Depending on availability of well characterized antibodies, stemness can be diagnosed immunohistochemically in sections of biopsies. This has already been done for TNBC using a novel monoclonal antibody against nNav1.5 [48, 66] or markers of EMT [67].

A particularly interesting approach would be to focus of autoantibodies (autoAbs) to embryonic proteins likely to be related stemness. In this situation, the immune system generates antibodies to an antigen that is deemed ‘foreign’. In turn, these autoantibodies can give rise pathophysiological syndromes by binding to matching proteins. Some of these ‘embryonic proteins’ are neuronal since aggressive carcinomas often express characteristics usually found in neurones and the associated consequences are known as “paraneoplastic neurological syndromes” (PNS) [e.g. 68]. A well-known PNS is the Lambert-Eaton Myasthenic Syndrome in small-cell lung cancer (SCLC) where the immune system generates autoAbs to voltage-gated sodium and calcium channels as well as ionotropic acetylcholine receptors [69]. These autoAbs manifest themselves by impacting neuromuscular junctions. A similar phenomenon also occurs in breast cancer in response to expression of neonatal splice variant of the VGSC subtype, Nav1.5 (nNav1.5). The latter was shown previously to occur in TNBC and drive the metastatic process [45, 46, 70]. Thus, Rajaratinam et al. reported that autoAbs to nNav1.5 can be detected in serum of patients at levels that fell following treatment [71]. This phenomenon is most likely to be due to the highly antigenic nature of the spliced region of nNav1.5 [51]. Since this autoimmune response would be expected to be very early in the metastatic process (whilst the metastatic cancer cells are still confined to the primary tumour), autoAb’s can serve as ideal biomarkers of disease state and expected progression and thus facilitate treatment decisions.

### Therapeutics

There is a plethora of therapeutic opportunities for targeting CSCs, including combinatorial approaches, at various stages of development. Some of these are highlighted in the following sections. Importantly, CSCs and physiologic ESCs overlap in their molecular characteristics so extreme caution is needed in the selective targeting of the CSCs and their associated signalling mechanisms. The latter include Notch, Wnt/β-catenin and Hedgehog [72]. In addition, it is possible to apply ‘precision medicine’ testing drugs on individual patient-derived organoids [73].

#### Small Molecule Inhibitors

Li et al. used single-cell RNA sequencing to analyse the diversity of CSCs in TNBC and found a subpopulation that resembled the cells that form the breast tissue during pregnancy [74]. These CSCs expressed high levels of FXYD3 in combination with other CSC markers. FXYD3 is a regulatory component of the Na^+^/K^+^-ATPase pump and treating the cells with cardiac glycosides, which inhibit the pump, killed the CSCs selectively in vitro and in vivo. Clinically, FXYD3 + CSCs resisted neoadjuvant chemotherapy.

Another target worthy of note in relation to TNBC CSCs is transforming growth factor-β (TGF-β) (TGF-β) in chemotherapy-treated TNBC patients [75, 76]. Similar effects were seen in a mouse model treated with radiation [77]. In the latter study, application of an inhibitor of TGF-β1 (SB431542) was able to rescue and re-sensitize the cells to radiotherapy. These results are consistent with treatment-induced ‘stemness’ being reversed by TGF-β1 inhibitors.

The immune component of the TME in TNBC is also prone to epigenetic changes and some of these may be promoted by TGF-β [78, 79]. Accordingly, TGF-β inhibitors could be useful in treatment of TNBC. As far as we could ascertain, TGF-β inhibitors are not (yet) used in the clinic. However, some clinical trials have been considered [e.g. https://clinicaltrials.gov/study/NCT03834662].

#### Epigenetics

**Fig. 4:**
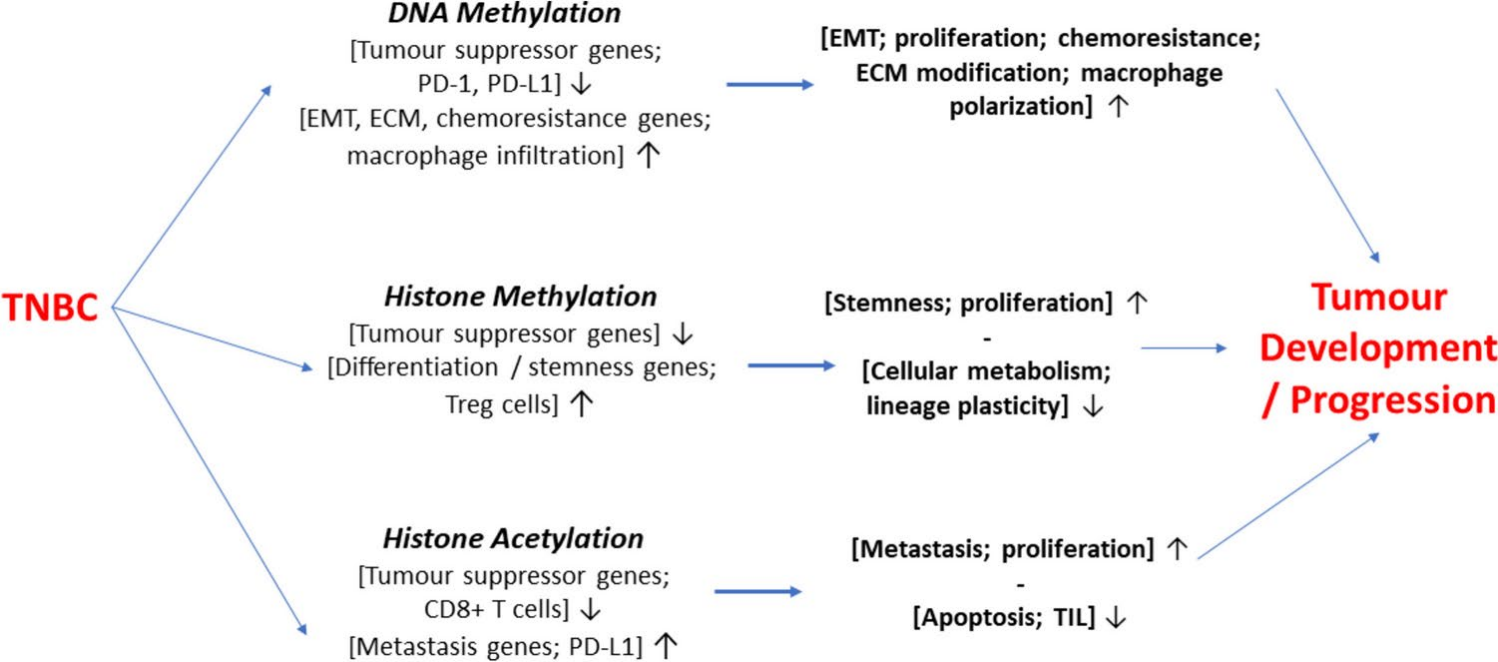
Epigenetic dysregulation of gene expression and cellular functioning in TNBC. Three epigenetic regulatory mechanisms are highlighted: DNA methylation, histone methylation and histone acetylation. There are several examples of molecular, biochemical and cellular consequences that follow from each, altogether regulating tumour development and/or progression. Upward and downward pointing arrows denote increasing and decreasing effects, respectively. Contents adopted from Huang et al. [16]

**Fig. 5:**
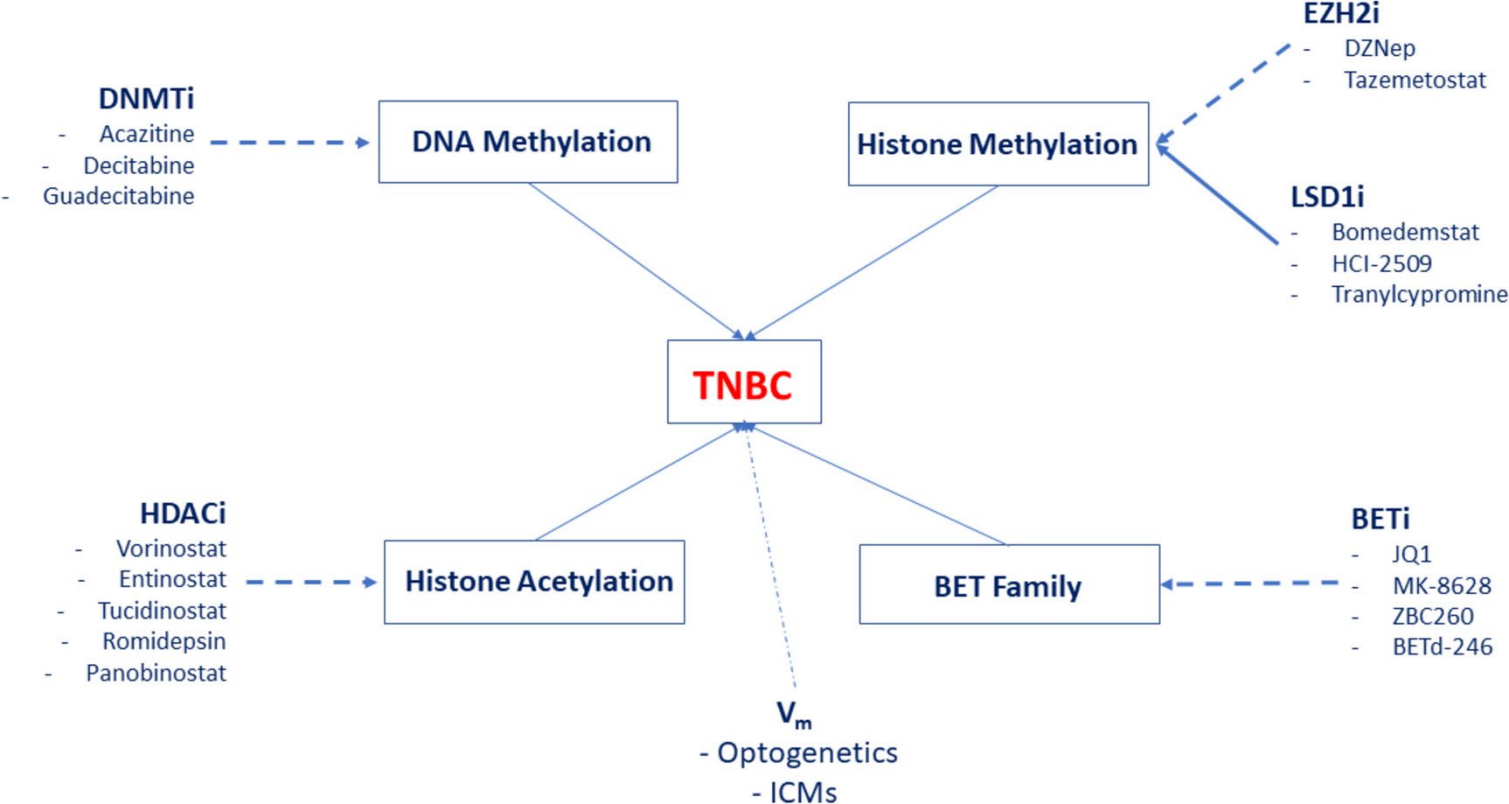
Examples of epigenetic drugs that can be used against TNBC. Four classes of drugs are shown: Inhibitors of DNA methyl transferase (DNMTi); inhibitors of histone deacetylase (HDACi); modulators of histone methylation—histone-lysine N-methyltransferase inhibitors (EZH2i) and lysine-specific demethylase inhibitors (LSD1i); inhibitors of bromodomain and extra-terminal motif proteins (BETi). Dotted and solid arrows denote inhibitory and stimulatory effects, respectively. In addition, regulation of membrane potential (V_m_) using optogenetics or ion channel modulators (ICMs) are indicated (undefined). Contents adopted and extended from Huang et al. [16]

Epigenetics plays a major part in the cancer process and recent evidence suggests that tumours can develop without any DNA mutation and this may be reversible [80]. Oncofoetal gene expression, common to dedifferentiation, is an epigenetic phenomenon which would imply that epigenetic modulators could also be employed against TNBC stemness. Possible epigenetic therapies of TNBC was discussed recently by Lehmann [81]. In another recent review, Zhou and Yu summarised the possible application of epigenetic drugs to TNBC as falling into 3 main areas (Figs. 4 and 5) [79].

1. DNA Methylation

This plays a key role in the pathogenesis of TNBC with the *BRCA1* gene appearing hypermethylated in over 50% of TNBC patients [82]. DNMT3A, a DNA methyltransferase, is frequently overexpressed in breast cancer, including TNBC, tissues, and its levels correlate with metastasis [83]. Inhibiting DNMT3A with low concentrations of decitabine suppressed 2D and 3D growth of TNBC cells [84]. Further DNA methylation events occur in the TME including upon genes involved in regulating the extracellular matrix (ECM) (Fig. 4).

2) Histone Methylation and Demethylation

These are mediated, respectively, by histone methyltransferases (HMTs) and histone demethylases (HDMs) which occur in TNBC and represent promising targets for its management [85]. In one example, pyruvate kinase M2 (PKM2) cascade was found to be hyperactivated and this would disrupt carnitine metabolism thereby hindering TNBC cell plasticity and, ultimately, worsening prognosis [86].

3) Histone Acetylation

Isoforms of histone deacetylases (HDACs) appear enriched in distinct cell types of breast cancer with HDAC3 particularly upregulated in TNBC [87]. Overall, the HDAC expression promotes dissemination of the cancer cells and EMT thereby leading to a negative association with survival [88]. Accordingly, HDAC inhibitors, including emerging new compounds, such as vorinostat (approved by the FDA) could be used against TNBC (Fig. 5) [89].

Serrano-Oviedo et al. adopted the TNBC-derived cell lines, MDA-MB-231 and BT549, and focused on bromodomain and extra-terminal domain (BET) family of epigenetic proteins to evaluate the effect of the inhibitor JQ1 on stemness [90]. Treatment of the cells (in spheroids) with JQ1 decreased surface expression of CD44 protein (a ‘classic’ stemness marker). The expression of two stemness-related genes (*ABCG2* and *RUNX2*) was found to be decreased by JQ1 which also inhibited the proliferation (via G0/G1 arrest), self-renewal and invasiveness of the cells. Structural analogues of JQ1 are being tested in clinical trials for a variety of cancers (JQ1 itself proved not suitable due to its short lifetime).

Epigenetic drugs can also be used to reverse drug resistance in TNBC. Indeed, a recent communication from the National Breast Cancer Foundation (USA) reported that the epigenetic (hypomethylating) drug decitabine could reverse the DNA methylation associated with chemoresistant TNBC and enhance the effectiveness of neoadjuvant chemotherapy on patient-derived models [91]; https://nbcf.org.au/project/innovative-epigenetic-therapy-for-optimising-triple-negative-breast-cancer-outcomes/#:~:text=Decitabine%2C%20an%20epigenetic%20therapeutic%20agent,responsiveness%20of%20chemotherapy%20in%20TNBC.

Another group of epigenetic modulators are non-coding RNAs, especially miRNAs and several of these ‘oncomiRs’ have been associated functionally with TNBC [92]. For example, transfection of miR-206 into TNBC cells downregulated VEGF, MAPK3 and SOX9 expression levels [93]. Loss of miR-214 enhanced the proliferative activity and epithelial-mesenchymal transition (EMT) altogether increasing the aggressiveness of TNBC [94]. miR-223 expression was low in TNBC CSCs and upregulating it re-sensitized the cells to TRAIL (tumour necrosis factor-related apoptosis-inducing ligand) [95]. Silencing miR-603 expression led to increased stemness of TNBC (eEF2K) and promoted growth, invasion, and disease progression [96]. In spite of this impressive range of functional effects impacted on TNBC by miRNAs, however, it is only possible to manipulate them using molecular techniques. For example, antisense RNA oligos can be optimized or crispr can be used to target specific miRNAs in cells. As ‘activators’, custom double-stranded synthetic miRNAs mimicking the action of endogenous miRNAs can be used [97]. This way, miRNA-targeted therapy is able to influence not only a single gene, but entire cellular pathways or processes.

Finally, we should note that, in common with current treatment strategies, epigenetic drugs can be combined effectively with other therapeutic modalities [98]. Possible synergistic combinations are with chemo/radiotherapy [99] and immunotherapy [100].

#### Immune Aspects and Biologics

Stemness of cancer including TNBC and the immune system are intimately associated, and immunotherapy is already a clinical reality. However, so far checkpoint inhibitor (CPI) (e.g. CAR-T) therapies have shown modest efficacy and only a small proportion (20–30%) of patients would benefit from this approach [101]. Consequently, emphasis has shifted to more personalised treatments driven by single-cell sequencing of the patient’s tumour. Emerging leads include the presence of high levels tumour infiltrating lymphocytes (TILs), PD-L1 expression and tumour mutation burden [102]. Another approach to boost the efficacy of CPI immunotherapy against TNBC is combination with other therapies (chemotherapy, radiotherapy or epigenetic therapies) [97]. Most recent evidence shows that an ‘artificial intelligence’ algorithm (“LORIS”) can be used to robustly predict patient outcomes with CPI immunotherapy using common clinical, pathologic and genomic features [103].

Importantly, the immune system offers various other forms of immunotherapy. First, TNBC-specific nucleic acid-based vaccines, such as DNA (as plasmids) and RNA (as mRNA) are being developed on a personalised basis [104]. Second, as noted in the “A Neuroscience Approach” section (with further details in the “Electroactive Agents and Electrotherapy” section), oncofoetal proteins can serve as neoantigens to enable production of cancer-specific monoclonal antibodies (mAbs). These can be used in several different ways. It is also possible that such mAbs can inhibit the functioning of some key protein by themselves. In that case, the mAb can be used directly as a drug. If the mAb is non-blocking, it can be adopted into ‘antibody-drug-conjugate’ (ADC) form and used for direct cancer cell / CSC killing [105]. Such mAbs can be used to coat drug-loaded nanoparticles and facilitate targeted delivery to tumours. Finally, oncofoetal proteins, even CEA, can form the basis of cancer vaccines [106].

The immunological aspects, including the immunotherapeutic potential, of the neonatal nature of the VGSC (nNav1.5) expression in major carcinomas (including TNBC) and cells of the immune system were discussed in detail earlier [51]. In one particular example, Dongre et al. (2020) showed that checkpoint inhibitors are more effective in the epithelial stage of tumours compared with mesenchymal [107]. Since VGSC is one of the promoters of EMT, checkpoint inhibition immunotherapy can be made more effective by combination with channel blockers.

#### Electroactive Agents and Electrotherapy

Although bioelectricity of cancer has a surprisingly long history, it is only in the 40 years or so that developments in molecular biology, electrophysiology and neuroscience have combined in series and in parallel to give us sufficient mechanistic insights and practical realities all the way to the clinic. There is now no doubt that bioelectric signals play a significant role in development and progression of cancer including TNBC. As regards stemness, several possibilities can be considered. First, there is the direct impact of the membrane voltage (V_m_). Although only some 10s of mV in absolute magnitude, resting V_m_ is equivalent to a trans-membrane voltage gradient of millions of volts per metre which can impact every protein in the cell membrane. Cancer cells, including the TNBC cell line MDA-MB-231 with stemness, and other proliferating cells have resting potentials of only ca. -20 to -25 mV compared with ca. -70 to -75 mV for quiescent cells such as resting neurones and muscles [108]. A range of evidence suggests that V_m_ hyperpolarization can promote (even be necessary and sufficient) for *differentiation* of a variety of stem cells [108, 109]. Thus, electromagnetic field-induced V_m_ hyperpolarization of human mesenchymal stem cells (MSCs) led to osteogenic differentiation and this occurred via voltage-dependent K^+^ currents accompanied by intracellular Ca^2+^ release [110, 111].

van Vliet et al. induced differentiation of human cardiomyocyte progenitor cells (CMPCs) by altering (hyperpolarizing) their membrane potential [109]. This was achieved by overnight culture in a low-potassium medium. Hyperpolarization led to increased intracellular calcium concentrations, activation of calcineurin signalling, increased cardiac-specific gene and protein expression levels and, ultimately, to the formation of spontaneously beating cardiomyocytes. A landmark study on human lung adenocarcinoma (A549) stem cells employed a more direct optogenetic (genetically coded voltage sensor) approach to manipulate the V_m_. It was thus found that facilitated anion transport (V_m_ hyperpolarization accompanied by acidification of intracellular pH) down-regulated various stemness biomarkers (EpCAM, CD44, CD166) [112]. Some anion transporter compounds produced similar effects and led to the elimination of CSCs. Thus, in both cases, simply hyperpolarizing the V_m_ was sufficient to induce differentiation. In a complementary approach, Stroth et al. developed an automated approach to track stem cell differentiation in an in vivo mouse model. Mouse ESCs were first stably transfected with channelrhodopsin-2 (ChR2) (yellow fluorescent protein) and purified by fluorescence activated cell sorting. As expected, illumination of the resulting ChR2-ESCs with pulses of blue light triggered inward currents, confirming the functionality of ChR2. These labelled ESCs retained the capability to respond to retinoic acid in vitro and differentiate into functional mature neurons, assessed by the presence of VGSC currents, action potentials, and expression of mature neuronal proteins and neuronal morphology. The differentiation process was enhanced by illumination, i.e. membrane *depolarization*! The ChR2-ESCs also survived when planted into the cortex but it was not clear if illumination affected the efficacy of the differentiation in vivo [113].

Second, there are various modulators of ion transporting proteins, especially ion channels, involved in generation of V_m_ and these can also affect stemness. Application of pharmacological agents (e.g. pinacidil and diazoxide) inducing short-term hyperpolarization of V_m_ led to MSC differentiation into both osteogenic and adipogenic lineages, with significant upregulation of the respective biomarkers [108]. Conversely, keeping the cells in a depolarized state, by increasing extracellular K^+^ or applying ouabain raised expression of MSC biomarkers and decreased osteogenic and adipogenic differentiation [108]. Furthermore, sufficient depolarization of a differentiated cell could induce reversal to a multi-potent progenitor [114]. The role of calcium-activated K^+^ (K_Ca_) channels in MSC differentiation was discussed earlier [52]. Importantly, there are several modulators of K_Ca_ channels that are clinically viable [115]. It is possible, therefore, that some of these could be employed against stem cells.

## Concluding Remarks

Stemness plays a significant role in the development and progression of aggressive cancers, including TNBC. By giving cancer cells extensive plasticity, it enables the cancer cells to adapt dynamically to their physical and biochemical environments including evading even the most aggressive treatments, and thus survive and propagate. At the same time, however, by differing in molecular terms from the rest of the heterogenous cellular makeup of tumours, CSCs could be deemed as having somewhat of a vulnerability. In this review, we dealt with these issues focusing on TNBC as an example of an aggressive cancer.

A major challenge is to distinguish CSCs from other stem cells, including ESCs, and target them whilst avoiding undesirable side effects. Modern molecular biology continues to give us detailed information about CSCs including their regulatory mechanisms. In particular, emerging (m)RNA-based techniques offer much promise also in terms of therapy. These encompass personalised cancer vaccines which are fast entering clinical trials [https://www.england.nhs.uk/2024/05/thousands-of-nhs-patients-to-access-trials-of-personalised-cancer-vaccines/]. Precision medicine enabling patient-specific treatments is clearly a way of the future and some of the treatments can be administered in combination to further increase efficacy, specificity and thus reduce potential undesirable side effects. Most recently, the field has gained novel impetus from neuroscience providing novel approaches such as bioelectric manipulation of CSCs and tumours including TNBC.

## Data Availability

Not applicable.
